# GEMO, a National Resource to Study Genetic Modifiers of Breast and Ovarian Cancer Risk in *BRCA1* and *BRCA2* Pathogenic Variant Carriers

**DOI:** 10.3389/fonc.2018.00490

**Published:** 2018-10-31

**Authors:** Fabienne Lesueur, Noura Mebirouk, Yue Jiao, Laure Barjhoux, Muriel Belotti, Maïté Laurent, Mélanie Léone, Claude Houdayer, Brigitte Bressac-de Paillerets, Dominique Vaur, Hagay Sobol, Catherine Noguès, Michel Longy, Isabelle Mortemousque, Sandra Fert-Ferrer, Emmanuelle Mouret-Fourme, Pascal Pujol, Laurence Venat-Bouvet, Yves-Jean Bignon, Dominique Leroux, Isabelle Coupier, Pascaline Berthet, Véronique Mari, Capucine Delnatte, Paul Gesta, Marie-Agnès Collonge-Rame, Sophie Giraud, Valérie Bonadona, Amandine Baurand, Laurence Faivre, Bruno Buecher, Christine Lasset, Marion Gauthier-Villars, Francesca Damiola, Sylvie Mazoyer, Sandrine M. Caputo, Nadine Andrieu, Dominique Stoppa-Lyonnet, GEMO Study Collaborators

**Affiliations:** ^1^INSERM, U900, Institut Curie, PSL Research University, Mines ParisTech, Paris, France; ^2^Service de Génétique, Institut Curie, Paris, France; ^3^Biopathologie, Centre Léon Bérard, Lyon, France; ^4^Hospices Civils de Lyon, Groupement Hospitalier EST, Bron, France; ^5^Gustave Roussy, Université Paris-Saclay, Département de Biopathologie et INSERM U1186, Villejuif, France; ^6^Département de Biopathologie, Centre François Baclesse, Caen, France; ^7^Institut Paoli Calmette, Département d'Anticipation et de Suivi des Cancers, Oncogénétique, Faculté de Médecine, Université d'Aix-Marseille, Marseille, France; ^8^Biopathologie, Institut Bergonié, Bordeaux, France; ^9^Service de Génétique, Hôpital Bretonneau, Tours, France; ^10^Service de Génétique, Centre Hospitalier de Chambéry, Chambéry, France; ^11^Service de Génétique Médicale et Oncogénétique, Hôpital Arnaud de Villeneuve, CHU Montpellier, INSERM 896, CRCM Val d'Aurelle, Montpellier, France; ^12^Service d'Oncologie Médicale, Hôpital Universitaire Dupuytren, Limoges, France; ^13^Université Clermont Auvergne, INSERM, U1240, Centre Jean Perrin, Clermont-Ferrand, France; ^14^Département de Génétique, CHU de Grenoble, Hôpital Couple-Enfant, Grenoble, France; ^15^Unité d'Oncogénétique, Centre Antoine Lacassagne, Nice, France; ^16^Unité d'Oncogénétique, Centre René Gauducheau, Nantes, France; ^17^Service d'Oncogénétique Régional Poitou-Charentes, Niort, France; ^18^Service Génétique et Biologie du Développement-Histologie, CHU Hôpital Saint-Jacques, Besançon, France; ^19^Université Claude Bernard Lyon 1, Villeurbanne, France; ^20^CNRS UMR 5558; Unité de Prévention et Epidémiologie Génétique, Centre Léon Bérard, Lyon, France; ^21^Institut GIMI, CHU de Dijon et Centre de Lutte contre le Cancer Georges François Leclerc, Dijon, France; ^22^INSERM, U1028, CNRS, UMR5292, Centre de Recherche en Neurosciences de Lyon, Lyon, France; ^23^INSERM, U830, Université Paris Descartes, Paris, France

**Keywords:** breast cancer, *BRCA1/2* mutation carriers, pathogenic variant (PV), DNA banking, genetic epidemiology

## Introduction

Women carrying a pathogenic variant (PV) in the *BRCA1* or *BRCA2* (*BRCA1/2*) genes are at high lifetime risk of developing breast cancer (BC) and ovarian cancer (OC), but estimation of the cumulative risk of cancer to age 70 years varies substantially between studies and populations. Initial estimations were obtained from selected high-risk families with multiple cases, such as those ascertained through the Breast Cancer Linkage Consortium used to identify disease loci (1). In the first retrospective studies conducted on such families, estimates for BC ranged from 40 to 87% for *BRCA1* PV carriers and from 27 to 84% for *BRCA2* PV carriers and estimates for OC ranged from 16 to 68% for *BRCA1* PV carriers and from 11 to 27% for *BRCA2* PV carriers (1–4). Recently, the largest prospective cohort conducted to date reported cumulative risks of BC to age 80 years of 72% for *BRCA1* PV carriers and 69% for *BRCA2* PV carriers (5). In the same study, cumulative risks of OC to age 80 years were 44% for *BRCA1* PV carriers and 17% for *BRCA2* PV carriers. Variation in cancer risks within or between BRCA1/2 families, with respect to age at diagnosis or type of cancer, can be explained by other genetic factors and/or lifestyle and reproductive factors (6–10). Genome-wide association studies (GWAS) conducted by the Breast Cancer Association Consortium (BCAC) have identified 172 common single-nucleotide polymorphisms (SNPs) associated with small increases in breast and/or ovarian cancer risk in the general population (11). A subset of these SNPs modifies the risk of breast and ovarian cancer risk for *BRCA1/2* PV carriers (12–14) but most of the variability has not been explained yet (15). Breast and ovarian cancer risks in *BRCA1/2* PV carriers might also vary according to the location of the variant and/or its origin (14, 16–19).

Genetic testing for *BRCA1* and *BRCA2* has been part of genetic counseling in European Union countries and North America since their discovery in the 90's, and has greatly improved recommendations about clinical management options and the most appropriate treatments. Nonetheless, both retrospective and prospective studies on large datasets of *BRCA1/2* PV carrier families are still very much needed to refine individual cancer risk estimates by considering other genetic and lifestyle/environmental factors, and they will also contribute to a better understanding of the correlation between mutant *BRCA1/2* alleles and phenotype. In particular, accurate age-specific risk estimates for the different types of cancer would be useful when choosing risk reduction strategies such as prophylactic bilateral mastectomy or salphingo-oophorectomy.

The Genetic Modifiers of *BRCA1* and *BRCA2* (GEMO) Group is the French multidisciplinary, collaborative framework for the investigation of genetic factors modifying cancer risk in Hereditary Breast and Ovarian cancer (HBOC) families segregating *BRCA1/2* PVs. Its primary aims are to contribute to large-scale national and international projects to identify genetic modifiers and to facilitate the translation of research results to the clinical setting. This is achieved by establishing a resource of blood DNA samples from individuals carrying a PV together with family and clinical data through the nation-wide network of cancer genetic clinics. Here we report on the progress of the GEMO study, the characteristics of the 5,303 actual participants and the prevalence and spectrum of *BRCA1/2* cancer-associated variants identified so far.

## Participants and methods

### Organization of cancer predisposition testing in france

GEMO investigators include molecular geneticists, clinicians, genetic counselors, and epidemiologists who are involved in the Genetic and Cancer Group (GGC), a consortium with support of UNICANCER whose objectives are to define optimal testing practices both in terms of genetic counseling and laboratory techniques, and to contribute to the estimation of individual's cancer risks (http://www.unicancer.fr/en/cancer-and-genetic-group). GGC has contributed to the national development of *BRCA1/2* screening tests and genetic consultations and, therefore improved management of subjects at high-risk of cancer.

Currently, there are 145 cancer genetic counseling units and 17 laboratories performing *BRCA1/2* testing (or panel testing of multiple cancer susceptibility genes) in France (see Supplementary Data for methods used by laboratories for PV identification).

Eligibility criteria for *BRCA1/2* testing according to the current national clinical guidelines are (i) at least 3 first or second degree relatives affected with breast or ovarian cancer in the same family branch, (ii) 2 first-degree relatives with BC, one of them having been diagnosed before age 41, or one before age 51 and the other before age 71, (iii) 2 first-degree relatives with BC, one of them being a male, (iv) 1 BC case before age 36, or before age 51 if triple negative tumor, (v) 1 case with bilateral BC, the first one before age 50, (vi) 1 male BC, (vii) 1 OC before age 71, or at any age if high-grade serous OC.

By 2016, 17,821 probands (i.e., the first individual tested in the family) were tested for *BRCA1/2*, and 1,670 (9.4%) were found to carry a PV. A similar number of probands carried a variant of uncertain clinical significance (VUS). A total of 6,417 relatives (essentially first-degree relatives of probands) underwent targeted screening tests and about 39% of them were found to carry the PV identified in the proband (http://www.e-cancer.fr).

### Ascertainment of GEMO participants

GEMO participants are from HBOC families ascertained prospectively through family cancer clinics and tested positive for a confirmed PV in *BRCA1/2*. The GEMO study was initiated in 2006 and is still ongoing. Initially, only female PV carriers aged 18 or older, affected or unaffected with cancer were invited to participate in the study by geneticists. Adult male PV carriers have been invited to participate since 2013. Today, GEMO involves 32 clinics and the 17 diagnostics laboratories from the GGC.

### Protocol, data collection, and database

The GEMO coordinating center was located at Centre Léon Bérard (Lyon) until September 2015 and is currently held at Institut Curie (Paris). All data and biospecimens are stored without personal identifiers. The GEMO case report form (CRF) includes information on participants' family history, gyneco-obstetrics risk factors (age at menarche, number of pregnancies, age at menopause), preventive surgery and tumor pathology (histology, grade, tumor size, hormone receptors status). Data on socio-demographic variables (age at inclusion, sex, ethnicity/population ancestry) and medical history of cancer (laterality, other cancer prior recruitment into study) are also collected.

Geneticists invite *BRCA1/2* PV carriers, whether affected with cancer or not, to participate in GEMO during the consultation informing them of their *BRCA1/2* positive test results. After completing the CRF with the participant, the geneticist sends it to the coordinating center, and requests that an aliquot of the blood DNA sample (at least 10 μg) that was used for genetic testing is shipped from the testing laboratory to the coordinating center. The study protocol is illustrated in Supplementary Figure 1.

Recently, an upgraded electronic database on FileMaker Pro 16 (FileMaker Inc., Santa Clara, California, USA) was developed to collate, manage and distribute core data and DNA samples, and to facilitate inter-operability with the GGC BRCA1/2 (ex-UMD-BRCA1/BRCA2) database (20) and that of the prospective cohort on *BRCA1/2* PV carriers GENEPSO (21).

### Ethics

The study is performed in compliance with the Helsinki Declaration and received a favorable review of the French National Committees for personal data protection in medical research (CCTIRS N°07223 and CNIL agreement N°1245228). GEMO has human ethics approval at all the participating institutions where subjects are recruited. All research projects making use of data and/or materials collected by GEMO are required to have independent ethical approval from their host institutions. Participants give written informed consent during genetic counseling sessions and understand that as a result of participation, personal details will be recorded and stored in a coded format on a database. They consent to samples of DNA material prepared from blood cells being stored in a central location and to de-identified information and samples being made available for scientifically and ethically approved research projects. Informed consent agreements signed by participants are kept in the clinics.

### Access to DNA samples and to family and clinical data

Investigators wishing to use the GEMO DNA collection and related clinical and family data submit a brief expression of interest to principal investigators (gemo@curie.fr) who then circulate the proposal to the GEMO steering committee with a 10-day opportunity given to highlight any major issues, especially duplication of, or complementarity to, existing projects. If favorably reviewed, a full application is then submitted and verified to ensure that sufficient resources to conduct the project exist, the amount of DNA requested is appropriate, and that the proposal has any required ethics approvals. When the project is accepted, a material transfer agreement and/or a data transfer agreement are signed between the coordinating center and the research institution of the applicant. DNA samples along with related data are sent to the applicants who commit to providing annual progress reports. To further enrich the GEMO resource, applicants are required to supply their research data to GEMO after publication, and/or 12 months after completion of their projects.

### Variant classification

The description of the genetic variants follows recommendations proposed by the Human Genome Variation Society (22). Variants are denoted using the cDNA reference sequences NM_007294.3 (*BRCA1*) and NM_000059.3 (*BRCA2*). Only carriers of a clear *BRCA1/2* PV are included in GEMO. PVs are defined as variants considered as pathogenic by the GGC (20), the Evidence-based Network for the Interpretation of Germline Mutant Alleles consortium (23), the Consortium of Investigators of Modifiers of *BRCA1/2* (CIMBA) (24) and/or published variants classified as pathogenic using multifactorial likelihood approaches (25, 26).

## Results

### Collection of DNA samples and data

As of April 2018, 5,303 participants with available DNA sample had been enrolled in GEMO. Participants included 3,087 *BRCA1* PV carriers (2,877 women and 210 men) and 2,216 *BRCA2* PV carriers (2,005 women and 211 men) belonging to 2,190 and 1,544 families, respectively. The mean number of participants per family was 1.4 (range: 1–11). For 600 families, DNA samples were collected from three or more family members. While no individuals in the dataset carried more than a single PV, four families segregated two PV in two branches of the family (family 1: *BRCA1:*c.5137del and *BRCA2*:c.2808_2811del; family 2: *BRCA1*:c.1480C>T and *BRCA1:*c.3839_3843delinsAGGC; family 3: *BRCA1*:c.3841C>T and *BRCA2*:c.4889C>G; family 4: *BRCA1:*c.4391_4393delinsTT and *BRCA2*:c.7680dup).

### Participants' characteristics

At inclusion, 56.3% of *BRCA1* female PV carriers were diagnosed with BC (mean age at diagnosis: 41.3, range 22–81), 18.3% were diagnosed with OC or fallopian tube cancer (mean age at diagnosis: 51.9, range 16–92) and 33.2% were free of these cancers (mean age at inclusion: 40.5, range 18–101). With respect to *BRCA2*, 61.1% of female PV carriers had BC (mean age at diagnosis: 43.6, range 21–90), 10.1% had OC or fallopian tube cancer (mean age at diagnosis: 57.9, range 31–99) and 32.9% were free of these cancers (mean age at inclusion: 42.1, range 19–91). Among the 421 male participants, 2.9% of *BRCA1* PV carriers and 6.2% of *BRCA2* PV carriers were diagnosed with prostate cancer at inclusion (mean age at diagnosis for *BRCA1*: 61.5, range 48–71 and 64.1, range 50–78 for *BRCA2*). Ten percent of males carrying a *BRCA2* PV had BC (mean age at diagnosis: 58.8, range 44–77) vs. none in male *BRCA1* PV carriers. Detailed characteristics of participants (probands and relatives) according to their cancer status are shown in Table 1. Parity, age at menarche and age at menopause (natural or artificial) for female PV carriers are shown in Supplementary Table 1. Female participants reported an average number of live births of 1.7 and a mean age at menarche of 12.9 years. No difference in parity or age at menarche was observed between women affected and unaffected with cancer, and no differences were observed between probands and relatives. Mean age at menopause (natural or artificial) was 45.7 and 47.8 years in *BRCA1* and *BRCA2* PV carriers, respectively. Information on prophylactic mastectomy or salphingo-oophorectomy is not systematically recorded in GEMO. However, based on available data, we identified 600 out of 4,882 female participants (12.3%) who had had bilateral or unilateral mastectomy. For 50 of them mastectomy was prophylactic as they had not developed BC at inclusion (1.0%). Among the 1,496 women (30.6%) who had had bilateral oophorectomy at inclusion, 1,005 (20.5%) had not developed OC or fallopian tube cancer and this surgery was likely prophylactic.

**Table 1:** Characterisitics of the GEMO subjects.

	***BRCA1* families**						***BRCA2* families**					
	**Probands**			**Relatives**			**Probands**			**Relatives**		
	***N***	**Mean age at inclusion, y (range)**	**Mean age of onset for first cancer, y (range)**	***N***	**Mean age at inclusion, y (range)**	**Mean age of onset for first cancer (range)**	***N***	**Mean age at inclusion, y (range)**	**Mean age of onset for first cancer (range)**	***N***	**Mean age at inclusion, y (range)**	**Mean age of onset for first cancer (range)**
**INDIVIDUALS AFFECTED WITH CANCER**												
Women (breast only)	1,154	46.8 (19–92)	40.3 (22–81)	240	47.5 (18–77)	41.1 (25–71)	949	49.4 (21–86)	42.5 (21–77)	193	52.4 (21–87)	45.3 (25–76)
Women (ovarian/fallopian tube^a^)	445	57.2 (33–80)	51.8 (25–86)	81	55.8 (22–72)	51.9 (16–92)	177	62.0 (37–84)	57.6 (31–84)	26	67.3 (44–100)	60.2 (41–99)
Women (breast and ovarian/fallopian tube)	186	59.9 (37–80)	46.0 (26–71)	38	57.4 (39–70)	46.0 (31–71)	72	63.2 (50–84)	49.1 (25–76)	11	71.6 (46–100)	61.3 (36–90)
Men (breast only)	0	–	–	0	–	–	16	62.2 (49–79)	58.4 (47–77)	2	70.0 (69–71)	63.5 (63–64)
Men (prostate only)	0	–	–	6	68.7 (58–78)	61.5 (48–71)	3	64.0 (57–77)	56.5 (50–63)	7	69.6 (61–78)	64.8 (53–78)
Men (breast and prostate)	0	–	–	0	–	–	2	68.0 (67–69)	60.0 (60–60)	1	76.0	53.0
Women (other cancer^b^)	4	46.5 (33–58)	41.8 (31–56)	3	34.7 (24–43)	32.0 (21–39)	6	57.3 (44–69)	50.7 (34–64)	5	55.2 (45–69)	43.6 (30–68)
Men (other cancer^b^)	5	66.8 (49–80)	62.0 (45–72)	6	71.0 (64–79)	65.4 (62–70)	3	53.0 (40–73)	51.7 (39–71)	4	56.8 (45–73)	50.0 (28–65)
**INDIVIDUALS UNAFFECTED WITH CANCER**												
Women	500	39.9 (18–83)	–	449	38.6 (18–86)	–	334	42.3 (18–80)	–	314	39.8 (18–91)	–
Men	78	47.6 (21–72)	–	117	51.6 (23–89)	–	52	49.4 (19–80)	–	122	48.2 (19–79)	–

a *May have had also breast cancer*.

b *Other than breast, ovary/fallopian tube, prostate cancer or basal cell carcinoma*.

Only 26.9% of participants self-reported their population ancestry/ethnicity. Among them, 90.8% were European, 3.5% were African, 0.3% were Asian and 4.1% were of other or mixed origin. Ashkenazi Jewish (AJ) ancestry was reported by 1.3% of participants.

### *BRCA1* and *BRCA2* variants

**Figure 1:**
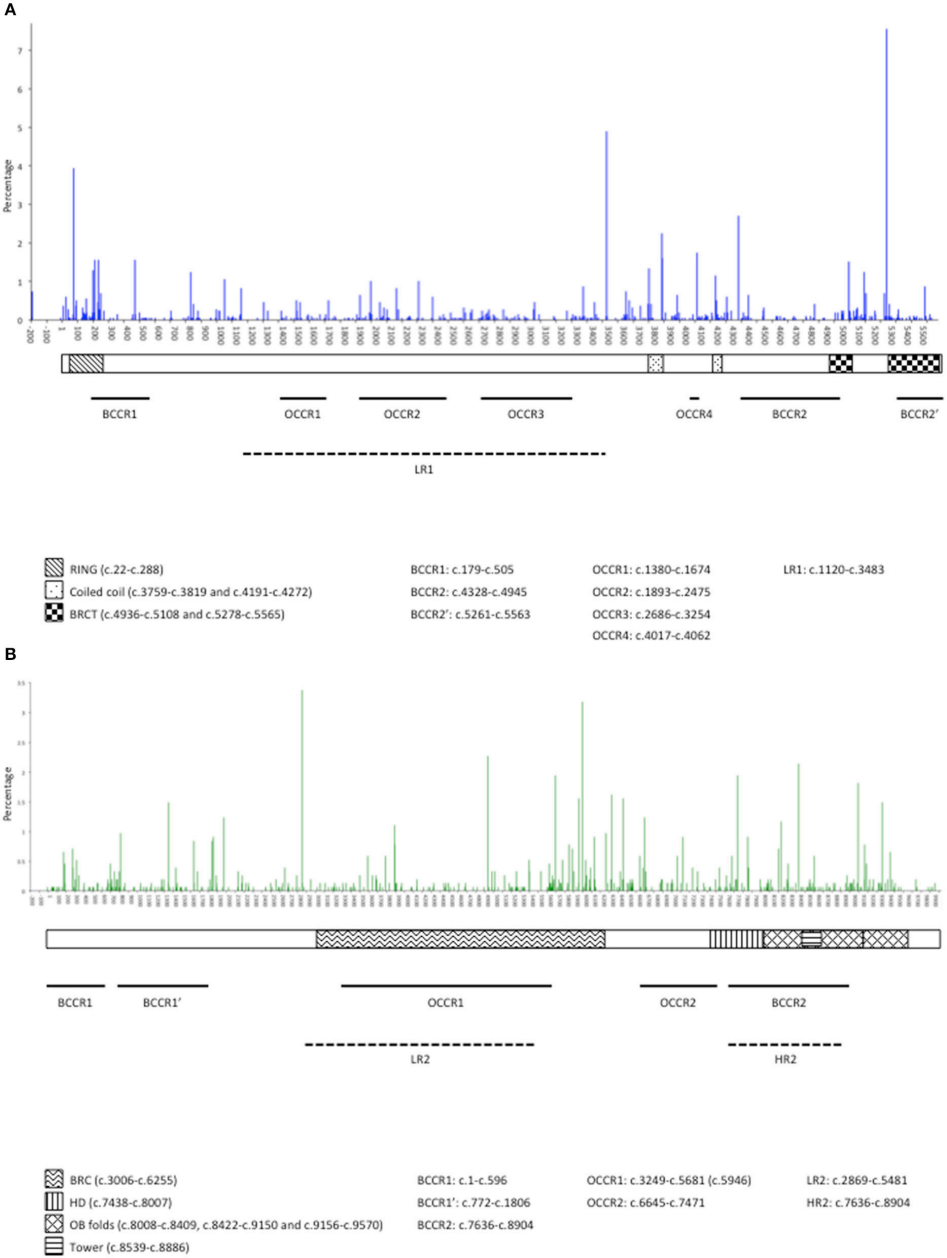
Distribution and occurrence of pathogenic variants along *BRCA1* **(A)** and *BRCA2* **(B)** in probands in the GEMO study. BRCA1 domains are: RING domain, Coiled Coil domains, BRCT (BRCA1 C-terminal) domains (14, 27). BRCA2 domains are: BRC repeats, helical domain (HD), OB fold binding domains, tower alpha (14, 28). Breast cancer risk regions: LR1, lower risk region in *BRCA1*, LR2 lower risk region in *BRCA2*, HR2, higher risk region in *BRCA2* (29); BCCR, breast cancer cluster region (14); Ovarian cancer risk regions: OCCR, ovarian cancer cluster region (14).

Currently, 506 *BRCA1* and 494 *BRCA2* unique PVs are described in the GEMO database. The number of families in which each PV was observed is shown in Supplementary Table 2 and the distribution of PVs across the gene sequences is shown in Figure 1. The five most common PVs accounted for 21.3% of all PVs in *BRCA1* and 14.9% of all *BRCA2* PVs. The most common *BRCA1* PVs were c.5266dup (7.5%) and c.68_69del (3.9%), originally described as founder PVs in the AJ population (30), the c.3481_3491del founder PV from North-Eastern France (4.9%) (31, 32), and the two common European PVs c.4327C>T (2.7%) and c.3839_3843delinsAGGC (2.2%) (33). The most common *BRCA2* PVs were c.2808_2811del (3.3%), c.5946del (3.2%), a Western European PV of AJ origin (34), c.4889C>G (2.2%), c.8364G>A (2.1%), c.5645C>A (1.9%), and c.7680dup (1.9%). There were 267 *BRCA1* PVs and 265 *BRCA2* PVs observed only once in GEMO.

### Representativeness of the GEMO population

The GGC database was designed to compile information on all *BRCA1/2* variants (pathogenic, neutral and VUS), except common polymorphisms, identified probands in the 17 French licensed laboratories (20). This database is therefore considered as the reference database for *BRCA1/2* variants in France. In June 2018, it contained PV from 6,385 BRCA1 and 4,839 BRCA2 families (Sandrine Caputo, personal communication), and about one third of the population recorded in the GGC database had been enrolled in GEMO. The distribution of PVs along the genes sequence in GEMO and the GGC *BRCA1/2* database overlaps (Supplementary Table 2), although a few variants were under-represented in GEMO reflecting a recruitment bias in the study due to the absence of participating cancer clinics in some regions (e.g., *BRCA1*:c.5260G>T is identified mostly in families from Western France). Other differences can be attributable to a different dynamics between the GEMO and the national registry (some PVs observed in GEMO had not been yet recorded in the GGC database).

## Discussion

Over 5,300 participants have been enrolled in GEMO to date, which provides an overview of *BRCA1*/2 PVs in a well-characterized sample of French counseled HBOC families. The GEMO resource is available to internal and external researchers who can apply for blood DNA and data for use in ethically approved, peer reviewed collaborative and interdisciplinary projects on the genetic epidemiology of cancer in BRCA1/2 families. Its overall goal is to facilitate the translation of research results to the clinical setting.

As an example, GEMO contributes massively to the CIMBA effort involving centers on six continents that have recruited *BRCA1/2* PV carriers with associated clinical, risk factors, and genetic data (24). GEMO is one of the three most important contributors to CIMBA projects in terms of number of samples, phenotypic and pathology data. In total, 2,868 subjects (53.9% of the GEMO population) had been genotyped using the iCOGS and/or the Oncoarray chips in the context of large-scale GWAS (35, 36). In brief, these international initiatives led to the identification of 26 and 16 SNPs associated with BC risk for *BRCA1* and *BRCA2* PV carriers respectively, and the corresponding numbers for OC risk are 11 and 13 (15). The combined effect of these SNPs, modeled as Polygenic Risk Scores (PRS) is currently being investigated to improve individualized cancer risk predictions. Other goals of the Consortium are to precise age-specific cancer risk estimates considering position and functional effects of the PV, family history of cancer and genetic and lifestyle/hormonal modifier risk factors in order to integrate findings on SNPs into the genetic counseling process. GEMO study collaborators co-authored 43 CIMBA publications. Publications and summary results for iCOGS SNPs are accessible via http://cimba.ccge.medschl.cam.ac.uk/.

At the national level, the GEMO group is aiming to develop specific PRS in the French counseled families in order to assess the clinical utility of incorporating such scores in risk prediction models. Indeed, improvement in the performance of such models for risk stratification and personalized decision-making (e.g., prophylactic mastectomy/salphingo-oophorectomy or frequency of BC screening) has important clinical implications. Efforts are also being made to render the GEMO database interoperable with other national databases including that of GENEPSO, which is a prospective cohort initiated in 1999, where *BRCA1*/2 PV carriers are followed over time to observed prospectively characteristics of subjects who develop either primary or secondary cancers (5). To date, about 1,400 individuals have been enrolled in both GEMO and GENEPSO.

Clinical management of healthy women with a *BRCA1*/*2* PV involves a combination of frequent screening, especially of the breasts, risk-reducing surgeries and possibly chemoprevention (37). For these women, important decisions include whether or not to undergo preventive mastectomy and the age at which to undergo risk-reducing salphingo-oophorectomy. These choices are invasive, have substantial side effects, and are associated with adverse psychological effects (38). It is therefore important to have precise estimates of associated age-specific cancer risks to provide optimal advices to women carrying a PV. Hence, women at particularly high risk or with a high risk of disease at early ages may benefit from early intervention, and women at lower risk may opt to delay surgery or chemoprevention.

## Author contributions

FL, FD, SM, and DS-L coordinated the GEMO study. CN, MiL, IM, SF-F, EM-F, PP, LV-B, Y-JB, DL, IC, PB, VM, CD, PG, M-AC-R, SG, VB, LF, BB, CL, and MG-V invited GEMO participants. CH, BB-dP, DV, HS, NM, LB, MéL, FD, and FL managed the DNA samples. NM, LB, MB, FD, FL, MaL, and YJ managed family and clinical data. NM, LB, MéL, FD, YJ, SMC, and FL curated the variants databases. FL and YJ analyzed the data. FL and NA wrote the paper. All authors read and approved the final manuscript.

### Conflict of interest statement

The authors declare that the research was conducted in the absence of any commercial or financial relationships that could be construed as a potential conflict of interest.

## Supplementary material

Supplementary Table 1 Age at menarche, age at menopause and parity for women affected and unaffected with cancer.

Supplementary Table 2 Unique pathogenic variants and number of families in which each variant was observed. Variants are called using the cDNA reference sequences NM_007294.3 (*BRCA1*) and NM_000059.3 (*BRCA2*).

Supplementary Figure 1 Protocol of the study.

Supplementary Data Methods used for identification of *BRCA1/2* pathogenic variants.

## Abbreviations

BC: Breast Cancer
BCAC: Breast Cancer Association Consortium
BRCA1: BReast CAncer 1
BRCA2: BReast CAncer 2
CRF: Case Report Form
CIMBA: Consortium of Investigators of Modifiers of BRCA1/2
GWAS: Genome-Wide Association Study
HBOC: Hereditary Breast and Ovarian Cancer
OC: Ovarian Cancer
PRS: Polygenic Risk Score
SNP: Single Nucleotide Polymorphism
VUS: Variant of Uncertain Clinical Significance.
