# Supplementary material for: GEMO, a National Resource to Study Genetic Modifiers of Breast and Ovarian Cancer Risk in BRCA1 and BRCA2 Pathogenic Variant Carriers
Source: Front Oncol. 2018 Oct 31;8:490. doi: 10.3389/fonc.2018.00490 (PMC6220051; doi:10.3389/fonc.2018.00490)
Supplement: Supplementary Table 1 — Age at menarche, age at menopause and parity for women affected and unaffected with cancer. [file Table_1.DOCX]

**Supplementary Table 1.** Age at menarche, age at menopause and parity for women affected and unaffected with cancer.

|  | *BRCA1* | | | *BRCA2* | | |
| --- | --- | --- | --- | --- | --- | --- |
| Parameter | Mean age at menarche, y (range) | Mean age at menopause (natural or artificial), y (range) | Parity | Mean age at menarche, y (range) | Mean age at menopause (natural or artificial), y (range) | Parity |
| Probands, unaffected (any cancer) | 12.9 (9-18) | 47.2 (27-68) | 1.6 | 13.0 (9-19) | 48.3 (32-58) | 1.5 |
| Relatives, unaffected (any cancer) | 12.8 (9-18) | 47.0 (32-58) | 1.4 | 12.9 (9-18) | 48.3 (37-61) | 1.4 |
| Probands, affected with BC only | 12.9 (9-18) | 44.8 (25-60) | 1.8 | 12.9 (9-18) | 45.8 (23-63) | 1.8 |
| Relatives, affected with BC only | 13.0 (9-17) | 44.5 (30-57) | 1.7 | 13.0 (10-18) | 46.1 (27-60) | 1.9 |
| Probands, affected with OC/fallopian tube | 12.9 (9-18) | 46.8 (32-59) | 2.1 | 12.9 (10-16) | 48.4 (35-61) | 2.1 |
| Relatives, affected with OC/fallopian tube | 13.1 (11-16) | 45.4 (38-55) | 2.2 | 13.0 (10-15) | 51.8 (44-57) | 2.4 |
