# Supplementary material for: GEMO, a National Resource to Study Genetic Modifiers of Breast and Ovarian Cancer Risk in BRCA1 and BRCA2 Pathogenic Variant Carriers
Source: Front Oncol. 2018 Oct 31;8:490. doi: 10.3389/fonc.2018.00490 (PMC6220051; doi:10.3389/fonc.2018.00490)
Supplement: Supplementary Figure 1 — Protocol of the study. [file Presentation_1.PPTX]

## Slide 1
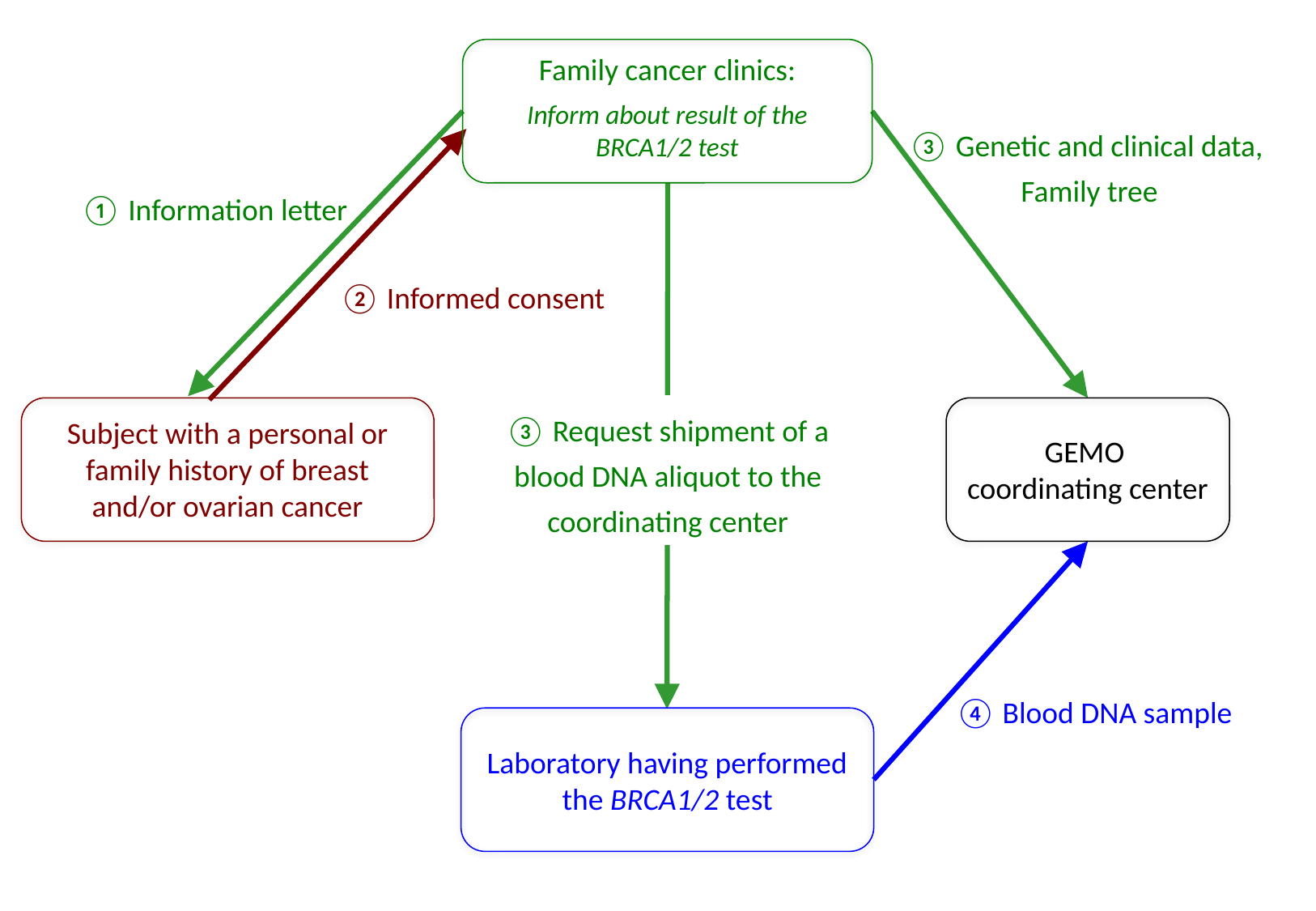

Family cancer clinics:
Inform about result of the BRCA1/2 test
 Genetic and clinical data,
Family tree
 Information letter
 Informed consent
 Request shipment of a blood DNA aliquot to the coordinating center
Subject with a personal or family history of breast and/or ovarian cancer
GEMO
coordinating center
 Blood DNA sample
Laboratory having performed the BRCA1/2 test
