# Supplementary material for: GEMO, a National Resource to Study Genetic Modifiers of Breast and Ovarian Cancer Risk in BRCA1 and BRCA2 Pathogenic Variant Carriers
Source: Front Oncol. 2018 Oct 31;8:490. doi: 10.3389/fonc.2018.00490 (PMC6220051; doi:10.3389/fonc.2018.00490)
Supplement: Supplementary Data — Methods used for identification of BRCA1/2 pathogenic variants. [file Data_Sheet_1.docx]

**Methods employed for identification of *BRCA1/2* pathogenic variants**

The full coding sequence and exon-intron boundaries of the *BRCA1* and *BRCA2* genes were screened using various approaches depending on the year of analysis, based on prescreening (denaturing Gradient Gel Electrophoresis (DGGE), Single-Strand Conformation Polymorphism (SSCP), Denaturing High Performance Liquid Chromatography (dHPLC) (Wagner et al., 1999), High-Resolution Melting curve analysis (HRM) (Coulet et al., 2010), or Enhanced Mismatch Mutation Analysis (EMMA) (Caux-Moncoutier et al., 2011)), and Sanger sequencing for point mutations. Screening for gross alterations was performed using large cDNA sequencing, Quantitative Multiplex PCR of Short fluorescent Fragments, Multiplex Ligation Probe Amplification analyses (Dehainault et al., 2004;Houdayer et al., 2004), qPCR-HRM (Coulet et al., 2010), or EMMA (Caux-Moncoutier et al., 2011). Next generation sequencing was used for more recent analyses (Collet et al., 2015).”

All cancer clinics from the GGC network are accredited by a national regulatory body, and molecular diagnostics laboratories are accredited by ARS (‘Agence Régionale de Santé’). They also got accreditation COFRAC ISO 15189 from the ‘Comité français d’accréditation’; <https://www.cofrac.fr/en/home>). They therefore have to follow the COFRAC Good Laboratories Practices for BRCA1/BRCA2 testing, and use similar technological methods, quality control procedures and variant classification. Those practices are in accordance with the European Molecular Genetics Quality Network (EMQN) best practice guidelines (<https://www.emqn.org>).

**References**

Caux-Moncoutier, V., Castera, L., Tirapo, C., Michaux, D., Remon, M.A., Lauge, A., Rouleau, E., De Pauw, A., Buecher, B., Gauthier-Villars, M., Viovy, J.L., Stoppa-Lyonnet, D., and Houdayer, C. (2011). EMMA, a cost- and time-effective diagnostic method for simultaneous detection of point mutations and large-scale genomic rearrangements: application to BRCA1 and BRCA2 in 1,525 patients. *Hum Mutat* 32**,** 325-334.

Collet, A.T., J.; Girard, G.; Dubois D’enghien, C.; Golmard, L.; Deshaies, V.; Lermine, A.; Laugé, A.; Moncoutier, V.; Lefol, C.; Copigny, F.; Dehainault, C.; Tenreiro, H.; Guy, C.; Abidallah, K.; Barbaroux, C.; Rouleau, E.; Servant, N.; De Pauw, A.; Stoppa-Lyonnet, D.; Houdayer, C. (2015). Pros and cons of HaloPlex enrichment in cancer predisposition genetic diagnosis. *AIMS Genetics* 2**,** 263-280.

Coulet, F., Pires, F., Rouleau, E., Lefol, C., Martin, S., Colas, C., Cohen-Haguenauer, O., Giurgea, I., Fajac, A., Nogues, C., Demange, L., Hardouin, A., Lidereau, R., and Soubrier, F. (2010). A one-step prescreening for point mutations and large rearrangement in BRCA1 and BRCA2 genes using quantitative polymerase chain reaction and high-resolution melting curve analysis. *Genet Test Mol Biomarkers* 14**,** 677-690.

Dehainault, C., Lauge, A., Caux-Moncoutier, V., Pages-Berhouet, S., Doz, F., Desjardins, L., Couturier, J., Gauthier-Villars, M., Stoppa-Lyonnet, D., and Houdayer, C. (2004). Multiplex PCR/liquid chromatography assay for detection of gene rearrangements: application to RB1 gene. *Nucleic Acids Res* 32**,** e139.

Houdayer, C., Gauthier-Villars, M., Lauge, A., Pages-Berhouet, S., Dehainault, C., Caux-Moncoutier, V., Karczynski, P., Tosi, M., Doz, F., Desjardins, L., Couturier, J., and Stoppa-Lyonnet, D. (2004). Comprehensive screening for constitutional RB1 mutations by DHPLC and QMPSF. *Hum Mutat* 23**,** 193-202.

Wagner, T., Stoppa-Lyonnet, D., Fleischmann, E., Muhr, D., Pages, S., Sandberg, T., Caux, V., Moeslinger, R., Langbauer, G., Borg, A., and Oefner, P. (1999). Denaturing high-performance liquid chromatography detects reliably BRCA1 and BRCA2 mutations. *Genomics* 62**,** 369-376.
